# Differential biomarker expression of blood and lymphatic vasculature in multi-organ-chips

**DOI:** 10.1038/s41598-025-96367-y

**Published:** 2025-04-25

**Authors:** Jonas Jäger, Maria Thon, Katharina Schimek, Uwe Marx, Susan Gibbs, Jasper J. Koning

**Affiliations:** 1 Department of Molecular Cell Biology and Immunology, Amsterdam UMC location Vrije Universiteit Amsterdam, De Boelelaan 1117, Amsterdam, 1081HV The Netherlands; 2 Amsterdam Institute for Immunology and Infectious Diseases, Inflammatory Diseases, Amsterdam, The Netherlands; 3 TissUse GmbH, Berlin, Germany; 4 Department of Oral Cell Biology, Academic Centre for Dentistry Amsterdam (ACTA), University of Amsterdam and Vrije Universiteit, Amsterdam, The Netherlands

**Keywords:** Organ-on-chip, Organ model, Vascularization, Blood vasculature, Lymphatic vasculature, Endothelial cells, Tissue engineering, Biotechnology, Cell biology, Lymphatic system, Lab-on-a-chip

## Abstract

Since the blood and lymphatic endothelium regulates homeostasis and inflammation during health and disease, establishment of vascularized Organ-on-Chip platforms with blood and lymphatic endothelial cells (BEC/LEC) is a pre-requisite to further advance the field of tissue engineering. Here, we aimed to determine whether characteristics of BECs and LECs cultured under flow in a multi-organ-chip (MOC) are influenced by shear stress or inflammation. Dermis-derived primary BECs and LECs were used to endothelialize a MOC followed by culture for up to 14 days at lymphatic and blood flow rates. Under blood flow, both cell types changed morphology, aligned in flow direction, and showed close cell-cell contacts as in *in vivo* blood vasculature. Under lymphatic flow, neither BEC nor LEC aligned, and both showed a cobblestone-appearance with limited intercellular contacts similar to lymphatics. Cells retained their cell type-specific phenotype and cytokine secretion profiles. CCL21 expression in LECs was rescued by flow, but diminished again with TNFα exposure, together with the LEC-specific markers PROX1 and TFF3. Homeostatic cytokine secretion was higher in BECs, but the response to TNFα was more pronounced in LECs. Results indicate that BEC and LEC phenotype and cytokine secretion is mostly an intrinsic property with only morphology and CCL21 being influenced by flow.

**Supplementary Information:**

The online version contains supplementary material available at 10.1038/s41598-025-96367-y.

## Introduction

Over the last years, three-dimensional (3D) tissue engineering of human organs has increased tremendously^1,2^. These advanced in vitro models increase the predictive value since they better represent human biology when compared to animal models. Their use in disease modelling, drug discovery and safety testing is already being implemented and holds great promise to reduce animal testing. Nevertheless, reconstructing human organs in all their complexity is still a challenge.

Within the human body, although organs are physically separated, there is considerable inter-organ communication. This is mediated via the complex network of blood and lymphatic vessels and involves various signals (soluble factors, exosomes, cells, etc.) to maintain viability and homeostasis^3^. Blood vessels supply oxygen and nutrients to the tissue, while lymphatic vessels support the transport of interstitial fluids containing biomolecules away from the tissue thereby maintaining tissue fluid homeostasis.

Both vessel types also play crucial roles in regulating immune responses e.g., inflammation. Blood vessels mediate the entry of immune cells into the inflamed tissue by the production of chemokines, cytokines and the upregulation of cellular adhesion molecules which allow for the extravasation of immune cells such as neutrophils, monocytes, and T cells into the tissue^4,5^. Lymphatic vessels support the transport of soluble antigens, antigen presenting cells such as Langerhans cells (LC, in skin and mucosa) and dendritic cells as well as other immune cells to draining lymph nodes for immune surveillance and activation. Lymphatic malfunctioning contributes to several human diseases such as lymphedema which can lead to increased risks of tissue inflammation and fibrosis^6,7^. In addition, chronic inflammation can result in increased lymphatic vessel density in human skin and influence the course of inflammation^8^.

The inner layer of both vessels consists of endothelial cells (ECs) that through adherens junctions, such as vascular endothelial cadherin (VE-cadherin), and tight junctions, such as CLDN5 and ZO-1, form a barrier between the circulation and the tissue^9^. ECs within blood vessels typically have tight intercellular contacts and align in the direction of flow to create a barrier between the peripheral blood and surrounding tissues, whereas lymph vessels have a cobblestone appearance with weak intercellular contacts to enable passive diffusion of interstitial fluid from the tissue into the initial lymphatics. As capillaries progressively merge, the lymph enters the collecting lymphatics with tighter cell-cell junctions, where it is propelled by muscle movement^10,11^. These ECs are highly heterogeneous depending on vessel type and location within the body^12,13^. Although their cell fate is determined during development^13^, a certain level of plasticity remains since environmental changes can influence lineage commitment. It has been shown that flow influences cell signaling, can induce morphological changes and even reprogram ECs^14,15^.

ECs of blood and lymphatic vessels can be identified by general cell surface markers such as CD31 (PECAM-1) and VE-cadherin (CD144) as well as by von Willebrand factor (vWF) located in endothelial specific organelles^12^. SOX18 and COUPTF2 are important transcription factors for both blood (BEC) and lymphatic endothelial cell (LEC) specification^16–18^. LECs can additionally be identified by nuclear PROX1, a transcription factor expressed in all LECs, and cell surface markers such as podoplanin (PDPN) and lymphatic vessel endothelial hyaluronan receptor 1 (Lyve-1)^19^. One of the hallmarks of LECs within skin tissue is the constitutive production of CCL21. This allows trafficking of immune cells from the tissue via lymphatic vasculature to the nearest draining lymph nodes for immune surveillance. Upon inflammation, LECs upregulate CCL21 to facilitate the induction of immune responses^20,21^. Previous studies have shown that upon static *in vitro* culture, LECs lose the expression of CCL21 which can be rescued by inflammatory stimuli^20^.

Most current human organ models are only used under static culture conditions. They do not incorporate vasculature and are therefore of limited use to study, for example, the migration of immune cells into or out of the tissue upon inflammation or the clearance of chemicals (such as drugs). This severely limits their relevance for pre-clinical safety and risk assessment or to study human diseases^22,23^.

To overcome these limitations, 3D-engineered tissues can be incorporated into organ-chips or multi-organ-chips (MOCs). These devices emulate human biology in vitro by enabling 3D organ tissues to be cultured under flow and have proven to be a valuable tool for further advancements of human organ models. Most of the models consist of a (simple) single tissue or organoids and lack complexity such as cross-organ communication as seen *in vivo*. This limits their relevance for systemic drug testing and systemic disease modelling such as inflammation and autoimmune diseases^24,25^. Integrating multiple organ models into MOCs further enhances simulation of human complexity^3^. However, most MOC models lack vascularization, both within the tissue and in the microfluidic channels. Since the blood and lymphatic endothelium regulate homeostasis and inflammation, establishment of endothelialized MOCs with BECs and/or LECs is a pre-requisite to further improve these human models.

The aim of this study was to determine BEC and LEC characteristics upon prolonged culture in a MOC and whether these characteristics are influenced by shear stress generated from different flow rates, representing high flow found in blood and low flow found in tissue draining lymph vessels, or whether the intrinsic properties of the cells are dominant. This is of importance when developing the next generation of MOC models and connecting different organoids through vasculature for e.g., improved drug testing platforms. Therefore, a method to isolate and generate pure populations of BECs and LECs from human dermis was developed. These BECs and LECs were used to endothelialize a MOC and cultured up to 14 days under blood or lymphatic flow rates. Histology, phenotype, and biomarker secretion profile upon culture at different flow rates under homeostatic and inflammatory conditions were determined for BECs and LECs in the endothelialized chips.

## Results

### Stable long-term culturing of BECs and LECs in chip upon different flow conditions

**Fig. 1:**
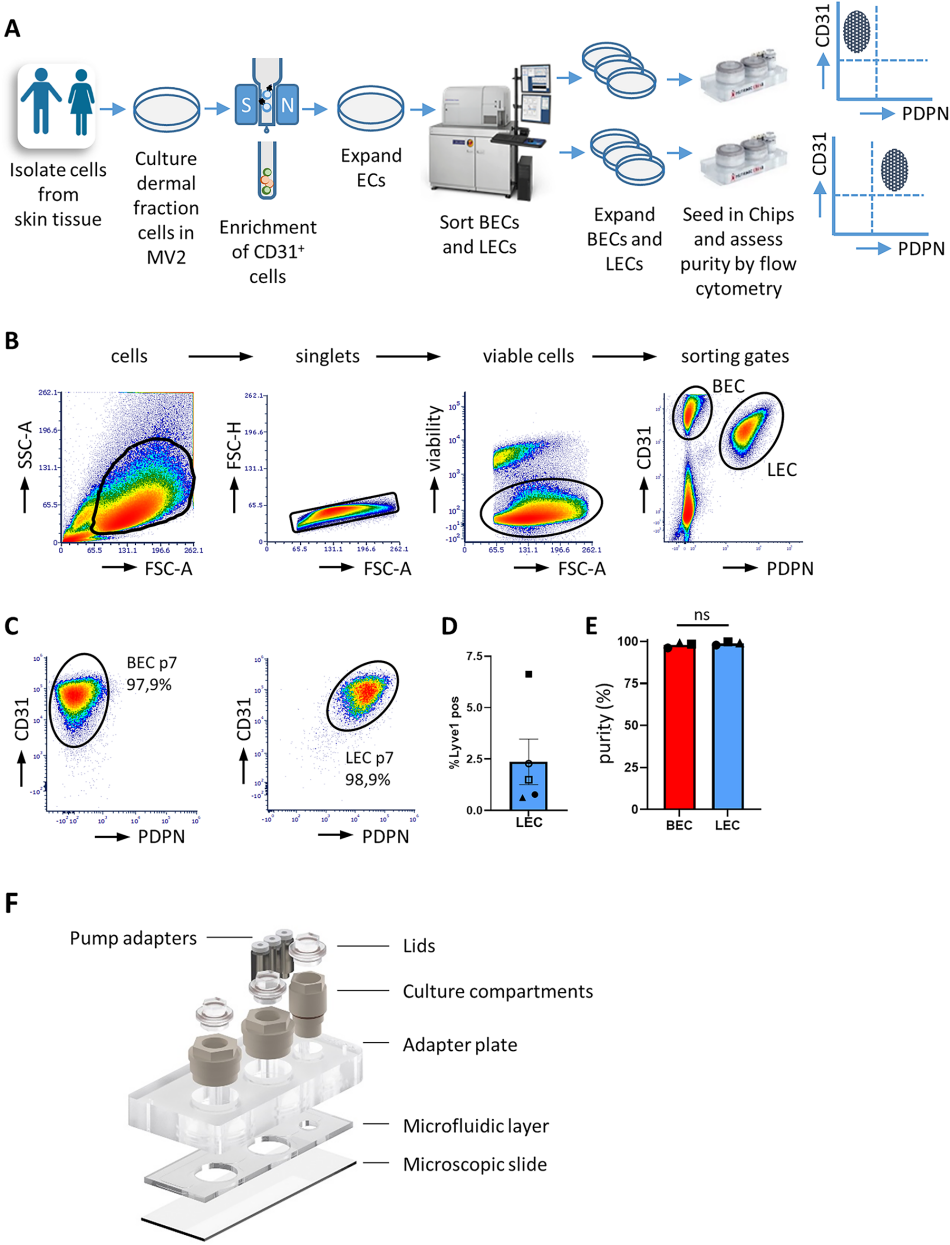
Isolation of primary blood and lymphatic endothelial cells from skin. (**A**) Workflow to obtain and expand pure populations of BECs and LECs from skin tissue for subsequent seeding in HUMIMIC Chips. (**B**,**C**) Representative example of (**B**) gating strategy for BEC and LEC isolation: cells, singlets, viable cells, ECs and BECs or LECs (the EC fraction was defined as CD31+, BECs as PDPN- and LECs as PDPN+), and (**C**) flow cytometry analysis of sorted cells upon prolonged culture periods until passage 7. (**D**) % of Lyve-1^+^ cells in LEC cultures. (**E**) Purity when seeding BECs and LECs in the chips was > 97.5%. (**F**) Explosion view of the HUMIMIC Chip3plus showing the microfluidic layer which is lined with BECs and LECs, and culture compartments used for sampling culture supernatant. MV 2: Endothelial Cell Growth Medium MV 2; EC: endothelial cell; BEC: blood endothelial cell; LEC: lymphatic endothelial cell. The data in (**E**) represent mean ± SEM; *n* = 3–5; ns = not significant; unpaired t-test. Symbols in bar graphs represent different experimental repeats with different donors (● repeat 1, ■ repeat 2, ▲ repeat 3, ○ repeat 4, □ repeat 5).

Since the number of ECs in the dermis of skin is low, a protocol for isolation and expansion of BECs and LECs to obtain high yields of pure populations was developed first (Fig. 1A). Direct enrichment for BECs and LECs after enzymatic digestion of primary skin tissue resulted in low cell numbers that poorly expand upon culture (data not shown). In contrast, short-term culturing of the dermal fraction first, followed by CD31 enrichment, expansion for one additional passage and subsequent sorting for BECs and LECs as described in our protocol resulted in highly pure populations of BECs (> 97.9% ± 1.59) and LECs (> 98.9% ± 0.97) (Fig. 1B). Both BECs and LECs maintained their CD31^+^PDPN^−^ (BEC) and CD31^+^PDPN^+^ (LEC) phenotype upon expansion and repeated passaging up to passage 7 (Fig. 1C). Analysis of Lyve-1 expression in expanded LECs revealed that most cells lack Lyve-1 expression (Fig. 1D). Purity before seeding the cells in the chips was > 97.5% (Fig. 1E).

**Fig. 2:**
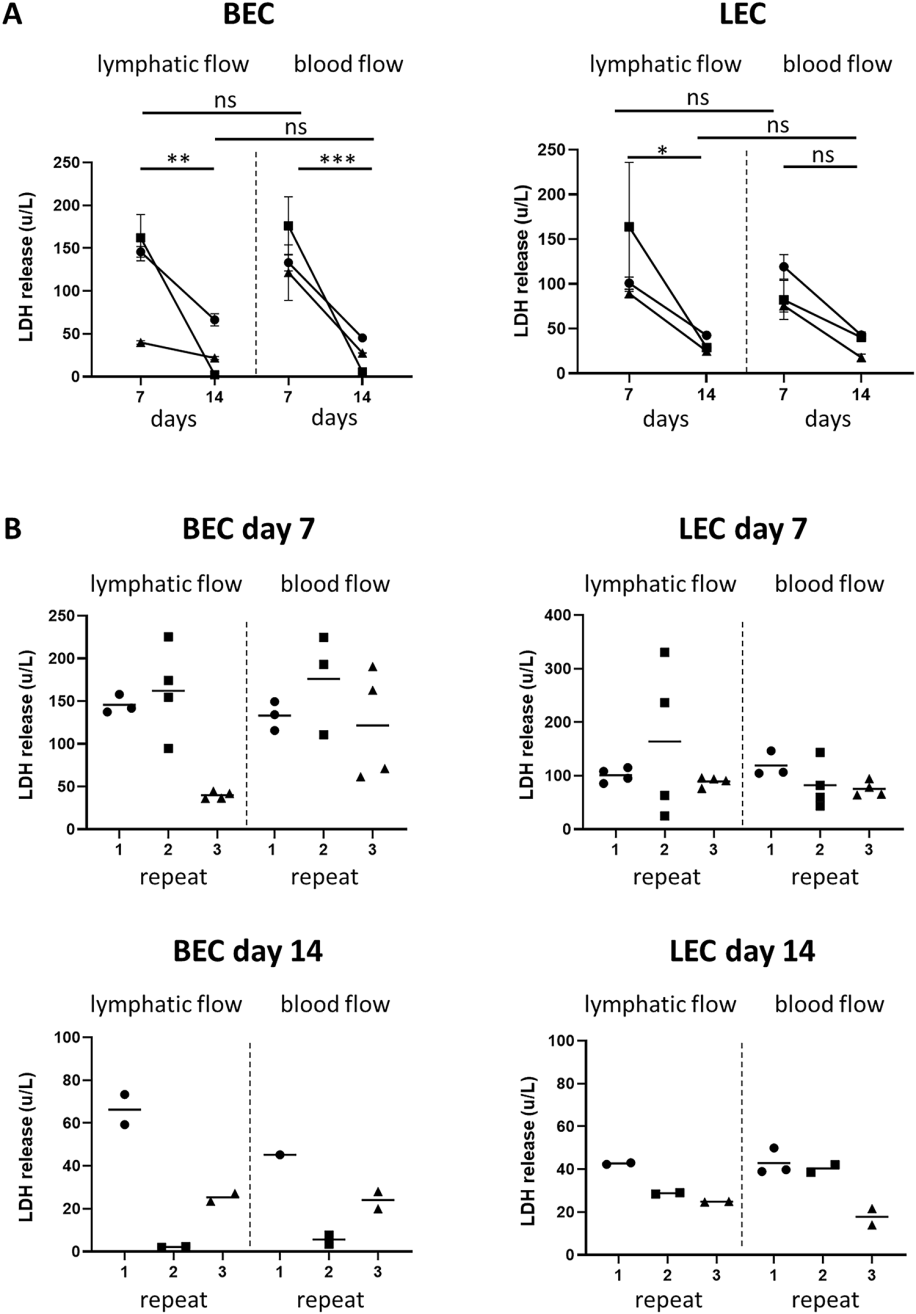
Cell viability in chips: LDH release (cell death) decreases over time. (**A**) LDH released by BECs (left) and LECs (right) in the culture medium on day 7 and 14 when cultured in different flow conditions. (**B**) Intra-experimental variation of LDH release of BECs and LECs cultured in lymphatic and blood flow at day 7 and 14. LDH: lactate dehydrogenase. The data represent mean ± SEM; *n* = 3, with 2 or more intra-experimental replicates (except repeat 1 BEC, blood flow, day 14); ns = not significant; *p < 0.05; **p < 0.01; ***p < 0.001; 2-way ANOVA. Symbols in bar graphs represent different experimental repeats with different donors (● repeat 1, ■ repeat 2, ▲ repeat 3).

To determine whether the phenotype of BECs and LECs was influenced by different flow rates, BECs and LECs were seeded into the HUMIMIC Chip3plus (Chip3; Fig. 1F) and cultured under blood or lymphatic flow for 7 and 14 days. The viability of the cultures was determined by assessing lactate dehydrogenase (LDH) release into the culture supernatant as high LDH release indicates leaky cell membranes and cell death (Fig. 2). On day 7, LDH release was detected in the culture supernatants and no significant differences in LDH levels were observed between BEC or LEC replicates when cultured under different flow conditions. At day 14, LDH levels decreased in all cultures, indicating less cell death and the establishment of stable culture conditions over time (Fig. 2A). Some experimental variation was observed between the three independent repeats which could be due to donor variability; however, little intra-experimental variation was observed between the replicates within a single experiment (Fig. 2B).

### Different flow conditions affect BEC and LEC morphology but not their phenotype

**Fig. 3:**
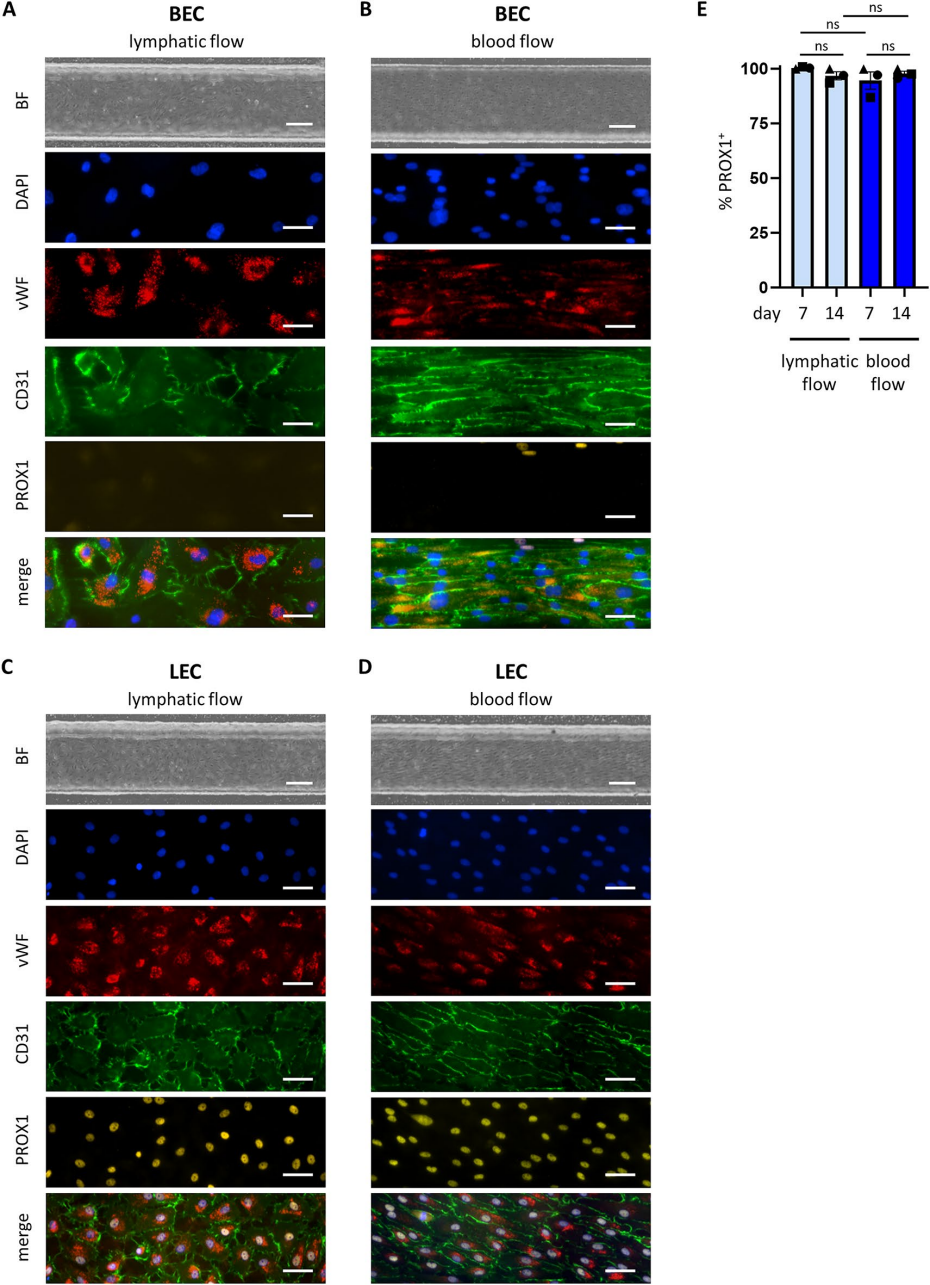
Flow changes morphology, but not phenotype of BECs and LECs. (**A**–**D**) Bright field overview and immunofluorescent stainings of endothelialized channels with BECs (**A**,**B**) or LECs (**C**,**D**) cultured under blood and lymphatic flow. (**E**) Percentage PROX1^+^ nuclei in LECs cultured in different flow conditions and culture periods. Scale bar in bright field images represent 200 μm, scale bar in immunofluorescent images represent 50 μm. Data in (**E**) represents mean ± SEM; *n* = 3, with 2 or more intra-experimental replicates; ns = not significant; 2-way ANOVA. Symbol shapes represent experimental repeats with different donors (● repeat 1, ■ repeat 2, ▲ repeat 3).

To determine whether flow influences the morphological appearance and the phenotype of BECs and LECs, both cell types were characterized for the expression of vWF, CD31 and PROX1 under blood and lymphatic flow (Fig. 3). BECs cultured under lymphatic flow did not align in the flow direction (Fig. 3A), while BECs cultured under blood flow conditions aligned and elongated in the direction of the flow (Fig. 3B). Similarly, when LECs were cultured under lymphatic flow, they did not align in the flow direction (Fig. 3C), while LECs cultured under blood flow did align (Fig. 3D). Both EC types expressed CD31 and vWF. Furthermore, upon lymphatic flow, both BECs and LECs showed a similar loose cobblestone morphology characteristic for LECs with CD31 clustering at the sites of cell-cell contacts (Fig. 3A,C). In contrast, upon blood flow, both BECs and LECs displayed tight cell-cell contacts and a more linear CD31 expression (Fig. 3B,D).

To determine whether LECs cultured in flow retained their lymphatic signature over time, nuclear PROX1 expression was analyzed by fluorescence microscopy. Notably, LECs cultured under lymphatic flow as well as blood flow conditions expressed PROX1 (Fig. 3C,D). Upon quantification, > 95% of all nuclei remained positive for PROX1, regardless of the flow condition they were cultured in (Fig. 3E). The LEC purity obtained after 7 and 14 days cultured in flow was comparable with the purity before seeding the cells in the chips (compare Fig. 3E with Fig. 1D). Taken together, these results show that whereas different flow rates influence morphology, they do not influence the specific identity of the BECs and LECs. This indicates that the two cell types did not transition into the different phenotypes solely by the environmental cue of shear stress.

### Different biomarker profile of BECs and LECs

**Fig. 4:**
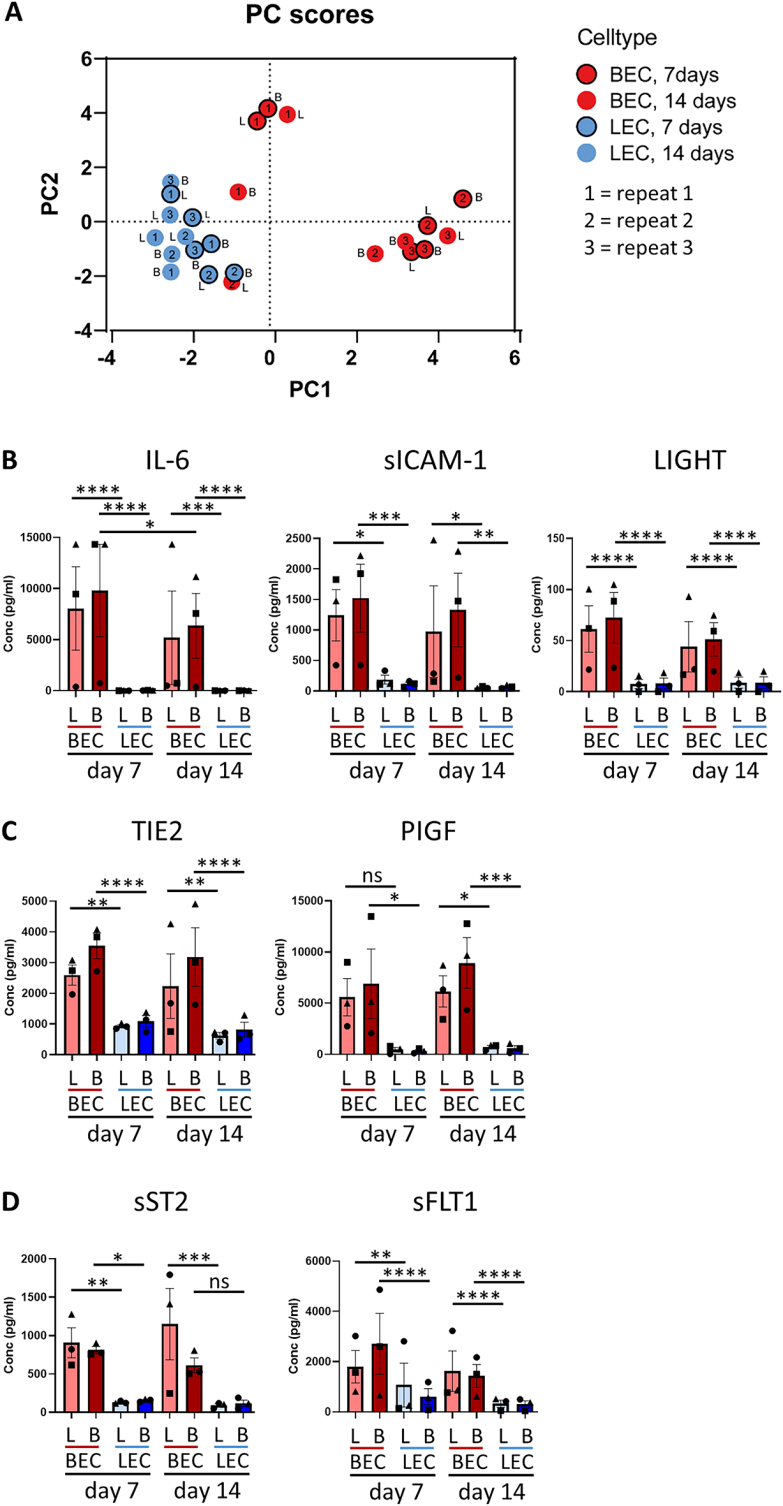
Distinct biomarker profiles of BECs and LECs remain unchanged with lymphatic and blood flow. (**A**) Principal component analysis of 16 biomarkers detected in supernatants of BECs (red) and LECs (blue) cultured under blood (B) or lymphatic (L) flow for 7 days (outlined) or 14 days. (**B**) Quantification of pro-inflammatory markers IL-6, sICAM-1 and LIGHT, (**C**) angiogenic markers TIE2 and PlGF and (**D**) anti-angiogenic markers/homeostatic markers sST2, sFLT1 in BECs or LECs cultured for 7 or 14 days under blood (B) or lymphatic (L) flow. PC: principal component. The data represent mean ± SEM; *n* = 3 with 2 or more intra-experimental replicates (except repeat 1 BEC, blood flow, day 14); *p < 0.05; **p < 0.01; ***p < 0.001; ****p < 0.0001; 2-way ANOVA. Symbols in bar graphs represent different experimental repeats with different donors (● repeat 1, ■ repeat 2, ▲ repeat 3).

Under physiological conditions, ECs release various soluble proteins which reflect their functional state. To analyze the secretome of LECs and BECs in the chips, multiple biomarkers were analyzed in culture supernatants. Principal component analysis revealed separation of BECs and LECs based on their secretome which was independent of flow conditions or culture duration (Fig. 4A). This indicates that flow conditions have no significant impact on secreted biomarkers. Except for IL-6, which decreased over time in BECs cultured in blood flow, no major differences were observed when BECs or LECs were cultured for 7 or 14 days. Pro-inflammatory markers IL-6, sICAM1 and LIGHT (Fig. 4B), pro-angiogenic markers TIE2 and PlGF (Fig. 4C), as well as homeostatic markers sST2 and sFLT1 (Fig. 4D), were secreted at higher levels in BECs when compared to LECs. Other pro-inflammatory (CCL2, MMP2, sVCAM1, TNFα and IL-18), angiogenic (TIE1) and homeostatic (Cystatin C, IL-10, IGFBP4) markers were detected at similar levels in both BECs and LECs, although the production levels of IL-10, IL-18 and TNFα were low and just above detection limits in both BECs and LECs (Supplementary Fig. S1). In summary, this data shows that BECs and LECs have their own distinct secretion profile which is not significantly influenced by different flow conditions.

### Comparison between flow and static culture: flow induces LEC-specific gene expression

**Fig. 5:**
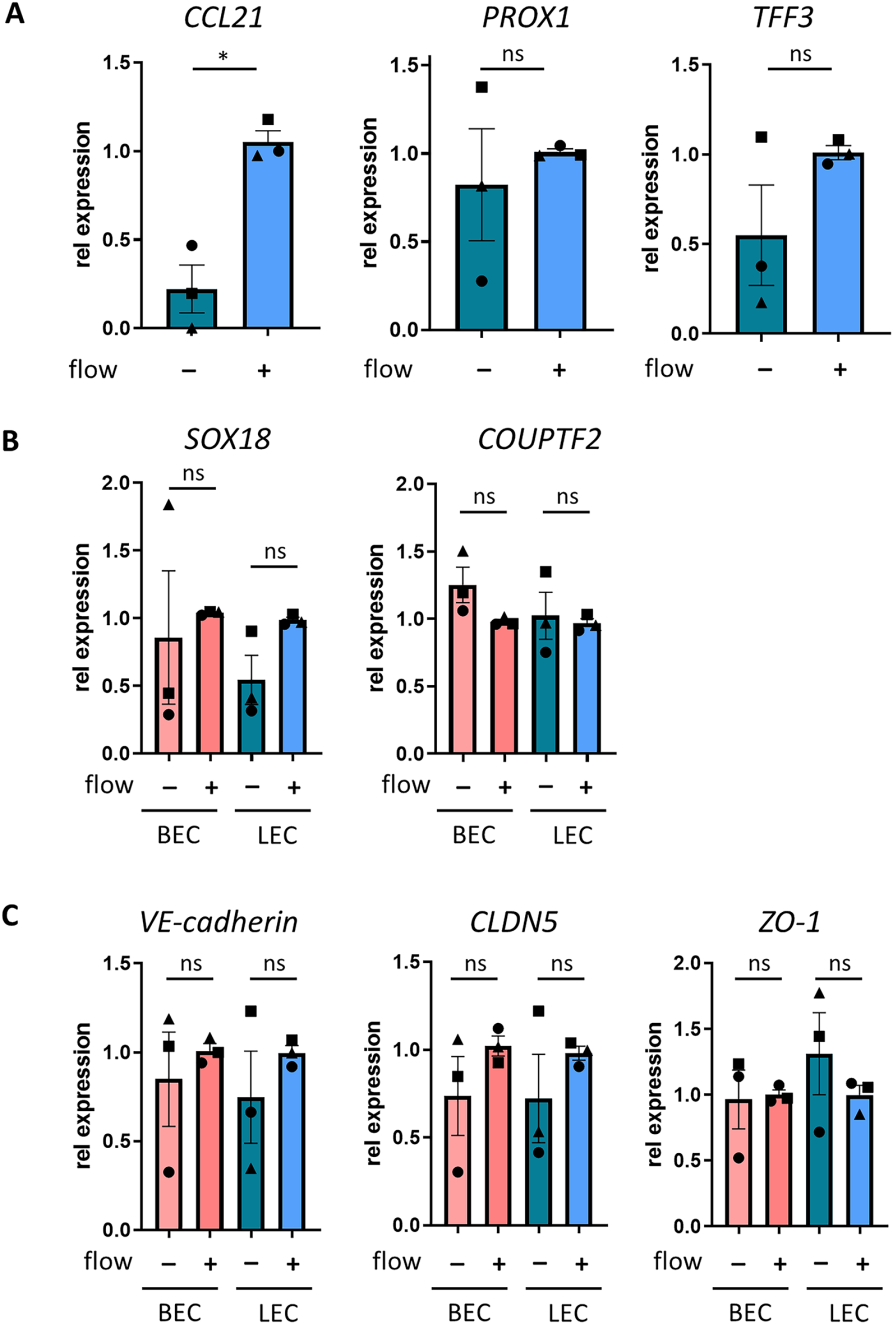
Flow upregulates LEC-specific gene expression. mRNA expression of BECs and LECs cultured without flow and 7 days with flow showing expression of (**A**) LEC-specific genes CCL21, PROX1 and TFF3 in LECs, (**B**) EC-specific genes SOX18 and COUPTF2, and (**C**) adherens and tight junction genes VE-cadherin, CLDN-5, ZO-1 in BECs and LECs. The data represent mean ± SEM; *n* = 3; ns = not significant; *p < 0.05; paired t-test. Symbols in bar graph represent different experimental repeats with different donors (● repeat 1, ■ repeat 2, ▲ repeat 3).

To determine whether flow influences BECs and LECs compared to conventional two-dimensional (2D) static culture conditions, key biomarkers were investigated at mRNA level (Fig. 5). Due to differences in culture design and media volumes, this comparison was not possible to do at protein level with secretomes. Furthermore, since flow conditions only affected cell morphology but not biomarker secretion, both cell types were cultured in their physiological flow rates, BECs in blood flow and LECs in lymphatic flow. When comparing morphology of BECs and LECs with and without flow, both BECs and LECs cultured under static conditions showed cobblestone-appearance similar to the morphology when cultured under lymphatic flow conditions (compare Fig. 3A-D with Supplementary Fig. S2). Importantly, culturing LECs for 7 days with lymphatic flow significantly increased mRNA expression of the LEC-specific chemokine CCL21 when compared to static cultures. Upon analysis of other lymphatic markers^26,27^, in 2 out of 3 repeats, increased PROX1 and TFF3 expression was observed when LECs were cultured under lymphatic flow compared to static cultures (Fig. 5A). When gene expression of SOX18 in BECs and LECs was compared between flow and static, again 2 out of 3 repeat experiments showed an increase in mRNA under flow (Fig. 5B). No differences in mRNA expression were observed for COUPTF2, adherens junctions VE-cadherin or tight junctions CLDN5 and ZO-1^9^ when comparing flow and static culture conditions (Fig. 5B,C). Even though some variability was observed between the different experiments (each with a different donor), variation between technical replicates within a single experiment (same donor) was low (Supplementary Fig. S3).

### Inflammation reduces LEC-specific marker expression and stimulates biomarker secretion

**Fig. 6:**
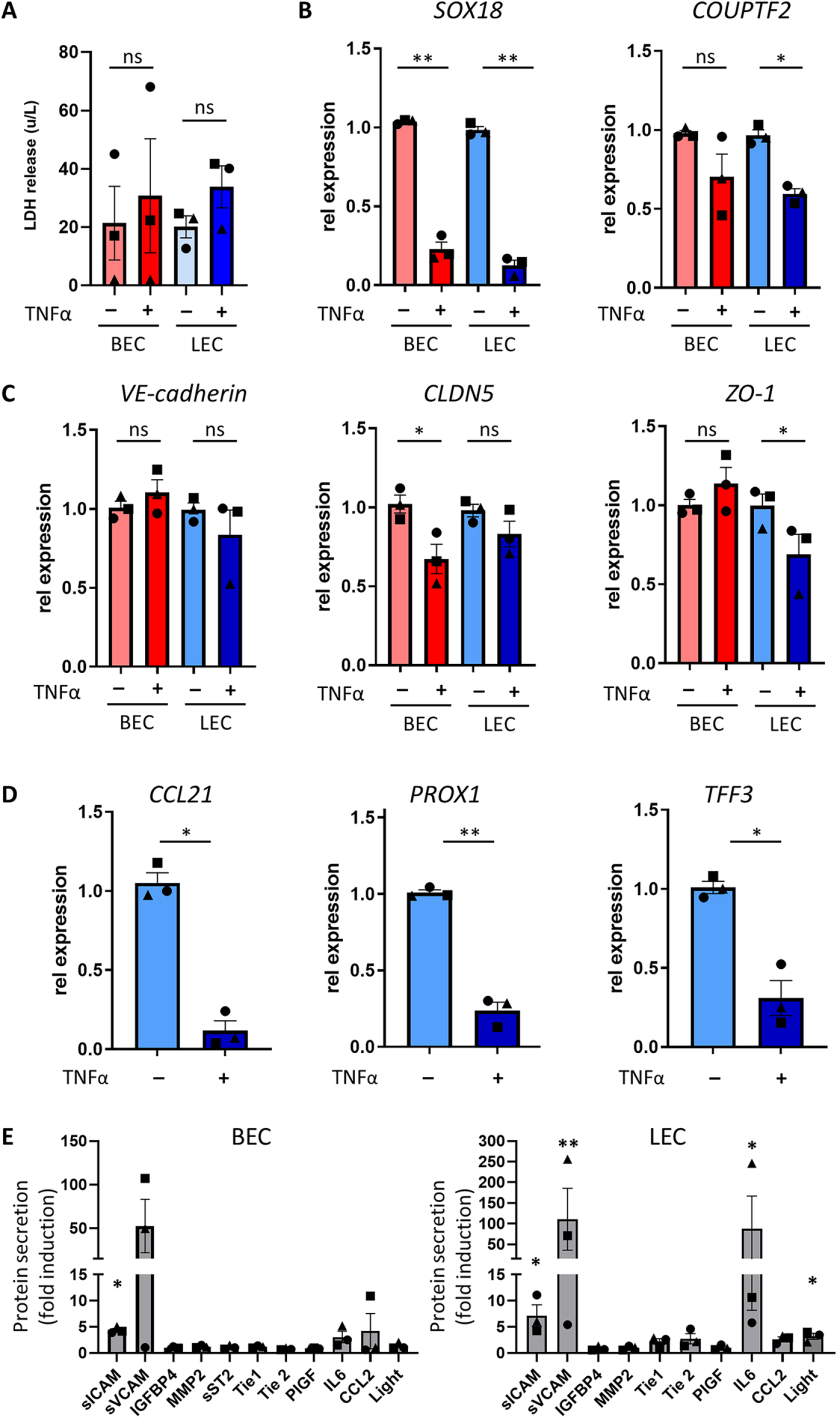
TNFα exposure diminishes BEC-, but especially LEC-specific gene expression and induces biomarker secretion. (**A**) LDH release of BECs and LECs with (+) or without (−) 24 h TNFα stimulation. Expression of EC-specific genes SOX18 and COUPTF2 (**B**), and adherens and tight junction genes VE-Cadherin, CLDN-5, ZO-1 (**C**) with or without TNFα stimulation. (**D**) Expression of LEC-specific genes CCL21, PROX1 and TFF3 in LECs. (**E**) Secreted biomarker fold change of BECs and LECs after TNFα stimulation, compared to unstimulated controls. The data represent mean ± SEM; *n* = 3; ns = not significant; *p < 0.05; **p < 0.01; paired t-test (**A**–**D**); 1-way ANOVA (**E**). Symbols in bar graph represent different experimental repeats with different donors (● repeat 1, ■ repeat 2, ▲ repeat 3).

BECs and LECs play important roles in inflammation. To identify how both cell types respond to inflammatory stimuli, chips were endothelialized with either BECs or LECs and cultured for 7 days to generate stable cultures. Both cell types were cultured in their physiological flow rates, BECs in blood flow and LECs in lymphatic flow. Subsequently, the cells in chips were exposed to TNFα for 24 h. No significant changes in LDH levels were observed upon TNFα exposure indicating that addition of TNFα was not cytotoxic (Fig. 6A). Upon stimulation with TNFα, mRNA expression of SOX18 was reduced in BECs and the expression of SOX18 and COUPTF2 was reduced in LECs (Fig. 6B). Stimulation with TNFα decreased CLDN5 expression in BECs and ZO-1 expression in LECs (Fig. 6C). Notably, TNFα stimulation reduced the expression of LEC-specific biomarkers CCL21, PROX1 and TFF3 in LECs (Fig. 6D) while in BECs, expression of these biomarkers was not detected (data not shown). Finally, the secretome of BECs and LECs was analyzed (Fig. 6E). When compared to unstimulated samples, a significant increase in sICAM-1 was observed in TNFα-stimulated BECs and LECs. In addition, in TNFα-stimulated LECs, increased secretion of sVCAM-1, IL-6 and LIGHT was observed, showing that LECs respond stronger to inflammatory stimuli than BECs.

## Discussion

The next challenge within the development of human representative* in vitro* models for drug testing and to study human physiology under homeostasis and inflammation is to incorporate both blood and lymph vasculature into the models. This will enable the infiltration of immune cells into organoids and organ models, and the migration of immune cells and metastatic tumor cells out via the blood and lymph vessels, respectively. However, such an advanced platform needs to be developed stepwise. In this current study, we describe the isolation and characterization of BECs and LECs from human dermis and show that, independent of whether they are cultured under flow or static conditions, they maintain their phenotype. Different flow rates did; however, influence morphology of ECs with blood flow stimulating cell alignment and tight cell-cell contacts which would be expected to influence barrier properties. Notably, low lymphatic flow rates increased the expression levels of lymph-specific genes compared to static cultures which is particularly important when considering the functionality of the lymphatics to draw immune cells into the vessel via chemoattractant gradients.

It has been shown that upon static *in vitro* culture of ECs, although pan-EC markers remain unchanged, expression of other cell surface markers can change. Dermal LECs are heterogeneous; initial lymphatics express both PDPN and Lyve-1 while pre-collecting and collecting vessels are Lyve-1^low^/PDPN^+^^28^. Our LECs expanded in 2D lack the expression of Lyve-1, suggesting that prolonged culture can reduce Lyve-1 expression as was shown before^29^ and that the cells obtained here can be characterized as collecting lymphatics.

In addition to phenotypic changes, the transcriptome of BECs and LECs changes upon micro-environmental cues which are related to cell functions^27,30^. For example, it has been shown that flow regulates CCL21 expression *in vivo*^31,32^. Interestingly, flow rescued CCL21 expression in LECs in our experiments. In addition, 2 out of 3 donors showed increased expression of the lymphatic markers PROX1 and TFF3, indicating enhanced functionality of LECs cultured in flow which is in line with previous findings^33^. Since LECs play a key role in the CCL21-mediated migration of immune cells out of the tissue into lymphatics and towards lymph nodes, this is of importance for future applications such as incorporation of LECs into immunocompetent engineered tissue to study the migration of immune cells in homeostasis and inflammation.

Flow rates and shear stress in the TissUse HUMIMIC Chips were characterized previously and adjusted to match physiological values in in vivo blood vasculature^34,35^. As shear stress has been described to be 10x lower in lymphatics when compared to blood vasculature with similar channel geometries^36^, we adjusted the flow parameters accordingly to mimic lymph flow in our experimental setup. Of note, with lymphatic flow rates, BECs and LECs displayed discontinuous button-like junctions comparable with initial lymphatics^11^. In line with the knowledge that EC-fate is determined early in development^13^, our results show that whereas blood and lymphatic flow rates influence the alignment and morphology of BECs and LECs, it does not influence the specific phenotype of the BECs (CD31^+^PROX1^−^) and LECs (CD31^+^PROX1^+^). This indicates that the two cell types are not able to transition into the different phenotypes by such an environmental cue. This is in contrast with findings by others who showed plasticity of human LECs when cultured under blood flow conditions for 8 h by downregulation of PROX1, one of the transcription factors involved in LEC differentiation^14^. However, we analyzed later time points in our study and did not look at PROX1 expression shortly after introducing blood or lymphatic flow. Most likely, stable cultures had been established after 7 or 14 days.

In ECs, several studies have shown the cells mechanosensitive properties by gene upregulation^37^ and post-translational protein modifications^38,39^ when comparing static to short-term flow cultures. In addition, short-term changes in wall shear stress^40^ and flow type (laminar to oscillatory)^41,42^ are involved in lymphatic valve formation. Others also described short-term effects of wall shear stress on ECs, but also an adaptive, stabilizing effect of gene expression 24 h after the onset of flow^43^. Additionally, it has been described that short-term (1 h) versus long-term (1 week) shear stress even had contradictory effects on endothelial monolayer permeability^44^. This emphasizes the importance of considering the perfused culture period when comparing such studies.

In our experiments, BEC and LEC vessels were perfused long-term (7 or 14 days) with constant flow rates to closely mimic physiological conditions. Different flow conditions did not influence the inflammatory secretome of BECs and LECs. In addition, we show that both EC types respond to the inflammatory stimulus TNFα with enhanced secretion of soluble proteins. Remarkably, this increase was more pronounced in LECs where a significant increase in sICAM, sVCAM, IL6 and LIGHT was observed. It is known that ECs increase the expression of membrane-bound adhesion molecules upon TNFα stimulation. In BECs, this is crucial for the process of adhesion, rolling and migration of immune cells out of the circulation into the tissue^4^. In LECs, membrane-bound expression of adhesion molecules might be involved in immune-cell migration out of the tissue into the lymphatics^45^. The specific role of increased levels of soluble adhesion molecules in LECs upon inflammation is unknown, but might help in migration of immune cells through lymphatics by regulating intra-lymphatic crawling and trafficking.

To our surprise, CCL21 gene expression was reduced in LECs upon stimulation with TNFα. It is known that CCL21 protein is stored intracellularly and can be mobilized to the cell surface upon TNFα stimulation^20,46^. In human skin, both CCL21 mRNA and protein expression were found to be upregulated in LECs in allergic contact dermatitis patients during an allergic inflammatory response^47^. However, others also found no upregulation of CCL21 mRNA upon *in vitro* stimulation of LECs with TNFα for 24 h^48^, suggesting that TNFα does not act directly on LECs to enhance CCL21 expression but rather facilitates chemokine secretion and that cellular interactions with immune cells or other ligands are required for *de novo* CCL21 synthesis. Other molecules such as LIGHT and Oncostatin M have been reported to regulate CCL21 transcription *in vitro*^48,49^. Although LIGHT secretion is increased in LECs upon TNFα stimulation in our experiments, this did not result in enhanced CCL21 levels.

Expression of SOX18, COUPTF2 and PROX1, three transcription factors involved in LEC-specification during development^15–18^, are also reduced upon inflammation with TNFα. This is in line with a previous study in mice where lymphatic marker genes were downregulated 24 h after skin inflammation^50^. Whether these markers remain downregulated and impact the LEC phenotype is unknown.

This study describes the isolation and expansion of BECs and LECs derived from primary human tissue, subsequent endothelialization of MOC channel structures, followed by their characterization under flow and inflammatory conditions. Although isolation of primary BECs or LECs from skin tissue has been described before^20,51,52^, our study focuses on the simultaneous isolation and expansion of BECs and LECs from the same donor in high numbers with extremely high purity which can be used for future tissue engineering applications. Furthermore, we describe the variation between the three different independent experiments (which combine technical variation and donor variation) and the variation between the intra-experiment replicates (same donor, different technical replicate performed in parallel). This is of importance when considering the reproducibility of such complex experimental designs e.g., for drug testing platforms, in the future. Even though we only performed 3 independent experiments showing rather high variation, the variation within the intra-experimental replicates was low. Consequently, donor variation must be considered, and such variation can be expected and appreciated when developing new drug testing platforms. Ideally, different donors should be compared within the same experiment to eliminant technical variation between experiments enabling only donor variation to be studied. However, scalability (sample size) is and will remain a limitation of the MOC system used here and of MOCs in general as these complex setups have low throughput due to model complexity, donor material availability and high cost.

Flow conditions did not influence the viability of cells. Although LDH levels were higher at day 7 when compared to day 14, this is caused by cell death of non-adherent ECs that occurs during the first days after initial seeding. These cells subsequently accumulate in the culture compartments and undergo apoptosis, a process where LDH is released into the supernatant. On day 14, we measured low LDH levels indicating that stable, viable cell culture conditions have been established.

Compared to BECs, LECs secreted lower levels of the proteins measured, but whether they secrete lower amounts of proteins in general was not assessed. Since the complete secretome of LECs is still largely incomplete, future studies unraveling it are of great interest to advance lymphatic vessel-on-chips.

Over the last decade, human tissue engineering has progressed from simple 2D monocultures to complex 3D organotypic systems which are integrated and combined in MOCs, to better recapitulate aspects of human tissues and human physiology over animal models. Since both BECs and LECs are important for tissue homeostasis and have complex control mechanisms upon inflammatory response, vascularization of engineered human tissue as well as endothelialization of microfluidic channels in MOC devices is important to further enhance applicability of organotypic models. It will enable studies on (1) the migration of immune cells in and out of the tissue in homeostasis and disease, (2) migration of immune cells within vessel-like structures and (3) crosstalk between ECs and 3D-engineered tissue. The methods and results presented in this study can be used to further advance the MOC-field by connecting functional blood and lymphatic vasculature to relevant healthy and diseased organ and organoid models to investigate human disease and safety testing.

## Materials and methods

### Isolation and expansion of dermal derived blood and lymphatic endothelial cells

Human skin from healthy donors was obtained as surgical waste upon abdominal dermolipectomy. The study was conducted in accordance with Declaration of Helsinki and the Dutch law (Medical Research Involving Human Subjects Act) as described in the ‘Code of Conduct for Health Research’ as formulated by COREON (https://www.coreon.org; Committee on Regulation of Health Research) and was approved by the institutional review board of the Amsterdam UMC. All subjects provided informed consent. Dermal ECs were isolated from skin tissue as follows (Fig. 1A); epidermal sheets were removed from the dermis after overnight incubation in dispase II (Sigma-Aldrich, St. Louis, MO, USA) at 4 °C followed by 10 min incubation at 37 °C. Remaining dermis was subsequently digested in collagenase (Gibco, Grand Island, NY, USA) for 2 h. To remove extracellular matrix, the cell suspension was filtered through a 40 μm cell strainer (Corning, Durham, NC, USA) and subsequently cultured at 37 °C, 5% CO_2_ in Endothelial Cell Growth Medium MV 2 (PromoCell, Heidelberg, Germany) until small endothelial colonies were formed. To obtain pure populations, ECs (passage 0) were harvested by trypsinization and enriched for CD31 using the human CD31 MicroBead Kit (Miltenyi Biotec, Leiden, The Netherlands) according to manufacturer’s instructions (Fig. 1A). After selection, cells were expanded in MV2 medium (Endothelial Cell Growth Medium MV 2; Promocell, Heidelberg, Germany). Subsequently, cells (passage 1) were harvested and sorted to obtain pure populations of BECs and LECs. Hereto, cells were incubated with Alexa Fluor-488 labeled anti-human podoplanin and PE labeled anti-human CD31 (both Biolegend, San Diego, CA, USA) and the viability dye Sytox™ Blue Nucleic Acid stain (Thermo Fisher Scientific, Waltham, MA, USA). Cells were sorted in BECs and LECs on a BD FACSAria™ Fusion Flow Cytometer (BD Biosciences, San Jose, CA, USA) with a 100 μm nozzle and low sorting speed (Fig. 1B). Sorted cells were collected in MV2 medium, centrifuged (300 g, 5 min, 4 °C), resuspended in MV2 medium and subsequently cultured for expansion. The purity of cells was assessed by flow cytometry as follows; cells were labeled with Alexa Fluor-488 labeled anti-human podoplanin, Lyve-1 PE (Novus Biological, Centennial, CO, USA) and Alexa Fluor-647 labeled anti-human CD31 and the viability dye Sytox™ Blue Nucleic Acid stain. Flow cytometric analysis was performed on an Attune Nxt (Thermo Fisher Scientific) and analyzed with FCS Express 6 or higher (De Novo Software, Pasadena, CA, USA).

### Endothelialization of HUMIMIC chips

HUMIMIC chips Chip2 24-well (Chip2) or Chip3plus (Chip3; TissUse, Berlin, Germany; Fig. 1E) were seeded with either BECs or LECs using the injection kit Injectfit C2-24 (Chip2) or the Injectfit C3P (Chip3, both TissUse) as follows: prior to endothelialization, chips were flushed with PBS (Fresenius Kabi, Amsterdam, The Netherlands) and subsequently coated with 150 µl coating solution consisting of Collagen IV (1 mg/ml, Sigma Aldrich, Amsterdam, The Netherlands), fibronectin (0.5 mg/ml, Corning Life Sciences BV, Amsterdam, The Netherlands) and sterile water (Fresenius Kabi) in a ratio of 40:20:40 respectively. Injection was performed with low dead space syringes provided with the injection kit. Chips were incubated overnight at 37 °C, 5% CO_2_. Next day, chips were flushed twice with PBS, filled with MV2 medium, and placed at 37 °C, 5% CO_2_ until use. 100 µl cell suspension in a concentration of 30 × 10^6^ cells/ml was injected in the chips. To attach cells to the channel walls, chips were subsequently incubated at 37 °C, 5% CO_2_ upright for 1 h, 2 h upside down, and 1 h upright again. Afterwards, injection fittings were replaced with cell culture compartments (TissUse) and filled with MV2 medium (500 µl per 24-well compartment, 300 µl per 96-well compartment). Cells were cultured continuously under blood flow (500 mbar, 0.5 Hz)^34,35^ or lymphatic flow (50 mbar, 0.5 Hz; 10x lower shear stress)^36^ by connecting them to the HUMIMIC Starter (TissUse). The next day, a complete medium change was performed to remove non-adherent cells. Total volume of medium in Chip3 was 1300 µl and 800 µl in Chip2. Medium was exchanged every 3–4 days as follows; for Chip3, 500 µl of medium was collected via the middle 24-well culture compartment and replaced with 500 µl pre-warmed MV2 (Fig. 1E); for Chip2, 300 µl medium was collected via the 96-well culture compartment and replaced with 300 µl pre-warmed MV2. Every 3–4 days, culture medium was refreshed with new medium. The removed medium (culture supernatant) of each time interval was collected and, after LDH analysis, stored at − 20 °C for future cytokine analysis.

### TNFα stimulation

For TNFα stimulation, medium in the Chip2 was completely removed and, after LDH analysis, stored at − 20 °C. MV2 medium, supplemented with 10 ng/ml TNFα (R&D Systems, Minneapolis, MN, USA), was added to the culture compartments (300 µl in the 96-well compartment, 500 µl to the 24-well compartment) of one circuit of the Chip2. The other circuit of the Chip2 was supplemented with MV2 medium only. The chips were re-connected to the HUMIMIC Starter and incubated perfused for 24 h at 37 °C, 5% CO_2_.

### Cell fixation in HUMIMIC chips

Cells within the Chip3 were fixed for histological analysis. Cells in Chip2 were either fixed for histological analysis or lysed for mRNA analysis. For fixation, medium from the culture compartments was removed, stored as described above and chips were washed twice with PBS (300 µl / insert, 5 min under flow, 37 °C, 5% CO_2_). After washing, PBS was removed from the culture compartments. Then, 300 µl paraformaldehyde (PFA, 4% aqueous solution, EM grade, Electron Microscopy Sciences, Hatfield, PA, USA) was added to each culture compartment, incubated 10 min under flow and 10 min static. Hereafter, PFA was removed completely, and chips were washed once with PBS containing 0.02% NaN_3_ and under flow for 5 min. Chips were stored at 4 °C until analysis.

### Immunofluorescent analysis

Cells in chips were stained as described earlier^34^. For immunofluorescent staining of PROX1 and vWF, chips were pre-treated with 0.2% PBS/Triton. Next, chips were incubated with unlabeled rabbit anti-human PROX1 (Reliatech, Wolfenbüttel, Germany) and mouse anti-human vWF (clone F8/86, M0616, DAKO, Glostrup, Denmark) in PBS/Triton for 30 min under flow and incubated for 6 h static at RT. Next, chips were washed twice with PBS/Triton for 10 min under flow, incubated with secondary antibodies: goat anti-rabbit 555 (A-21429) and goat anti-mouse 647 (A-21236, both Invitrogen, ThermoFisher Scientific, the Netherlands) in PBS/Triton under flow for 30 min at RT followed by static overnight incubation at 4 °C. The following day, chips were washed twice with PBS/Triton for 10 min under flow, followed by incubation with mouse anti-human, a directly labelled CD31 Alexa Fluor-488 (Biolegend) and DAPI (Invitrogen). Chips were stored at 4 °C until analysis, imaged on a Nikon Ti2 inverted microscope (Nikon Europe, Amsterdam, The Netherlands) and processed using Imaris 9.5.1 or higher (Oxford Instruments, Oxfordshire, UK). For each chip, the same region was imaged. To determine the percentage of PROX1 positive nuclei, the spots-selection tool of Imaris was used to select all DAPI^+^ and DAPI^+^PROX1^+^ nuclei.

### mRNA isolation and transcript analysis

For mRNA analysis, mRNA was isolated with the RNeasy Mini Kit (Qiagen, Hilden, Germany). Hereto, remaining medium in the chips was removed. To select for cells in the channels, cells in the culture compartments were removed mechanically. Chips were washed once with PBS under flow for 5 min. Subsequently, 150 µl lysis buffer was added to each culture compartment and incubated under flow for 5 min followed by 5 min static incubation at room temperature (RT). Lysing was monitored under the microscope. Lysed cells were collected, and mRNA was isolated according to manufacturer’s instructions and stored at -70 °C until further processing. mRNA from lysed cells was isolated using a RNeasy Mini Kit (Qiagen, Hilden, Germany) and cDNA synthesized using the RT2 First Strand Kit (Qiagen) according to the manufacturer’s instructions. Quantitative RT-PCR was performed on a QuantStudio™ 3 Real-Time PCR System (Thermo Fisher Scientific). The total volume of the reaction mixture was 10 µl and contained cDNA, 300 nM of each primer, and SYBR Green master mix (Applied Biosystems, Waltham, MA, USA). Primers are listed in Supplementary Table 1. Primers for GAPDH and HPRT are from Origene (OriGene Technologies, Rockville, Maryland). The comparative Ct method (ΔΔCt) with GAPDH and HPRT as the housekeeping genes was used to indicate relative changes in mRNA levels between samples. Relative mRNA levels of control-treated tissues were set at 1.0.

### Quantification of soluble analytes in culture supernatant

Profiling of soluble analytes in culture supernatants of BECs and LECs was performed using BioLegend’s LEGENDplex™ bead-based immunoassays. The human vascular inflammation panel 1-TC with v-bottom plate to detect Myoglobin, MRP8/14, NGAL, CRP, MMP-2, OPN, MPO, SAA, IGFBP-4, ICAM-1, VCAM-1, MMP-9, Cystatin C and human vascular inflammation panel 2-TC with v-bottom plate to detect sST2, sRAGE, TIE-2, sCD40L, TIE-1, Flt-1, LIGHT, TNFα, PlGF, IL-6, IL-18, IL-10, CCL2 (MCP-1) (BioLegend) was used. Legendplex immunoassays were performed according to manufacturer’s instructions, measured on an Attune Nxt (Thermo Fisher Scientific) and analyzed with the online available LEGENDplex™ Data Analysis Software Suite (BioLegend). Myoglobin, MRP8/14, NGAL, CRP, MPO, SAA and CD40L, MMP9, sRAGE and OPN were below detection limit.

### LDH analysis

Medium for LDH analysis was stored at 4 °C for a maximum of 7 days and LDH concentrations were determined using the Cytotoxicity Detection KitPLUS (Sigma Aldrich, Darmstadt, Germany) according to the manufacturer’s instructions. Absorbance measurements were performed in a microplate reader at 490 nm (Mithras LB 940, Berthold Technologies, Bad Wildbad, Germany).

### Statistical analysis

For both Chip2 and Chip3 experiments, three independent repeats with cells from different donors and intra-experimental duplicates were performed. Data are represented as mean with standard error of mean (SEM). Statistical analyses were carried out using GraphPad Prism 9.0 (GraphPad Software, La Jolla, CA, USA) as indicated in the figure legends.

## Electronic supplementary material

Below is the link to the electronic supplementary material.

Supplementary Material 1

## Data Availability

The raw data supporting the conclusions of this article will be made available by the corresponding author (jj.koning@amsterdamumc.nl), without undue reservation.

## Abbreviations

BEC: Blood endothelial cell
Chip2: HUMIMIC Chip2 24-well
Chip3: HUMIMIC Chip3plus
DC: Dendritic cell
EC: Endothelial cell
LC: Langerhans cell
LDH: Lactate dehydrogenase
LEC: Lymphatic endothelial cell
MOC: Multi-organ-chip
