# Supplementary material for: Differential biomarker expression of blood and lymphatic vasculature in multi-organ-chips
Source: Sci Rep. 2025 Apr 25;15:14492. doi: 10.1038/s41598-025-96367-y (PMC12032159; doi:10.1038/s41598-025-96367-y)
Supplement: Supplementary file 1 — Supplementary Material 1 [file 41598_2025_96367_MOESM1_ESM.pdf]

Supplementary Figure S1: Biomarker profile of BECs and LECs. Quantification of pro-inflammatory markers CCL2, MMP2, sVCAM-1, TNFα and IL-18 (A); angiogenic marker TIE1 (B); and anti-angiogenic markers/homeostatic markers Cystatin C, IL-10 and IGFBP4 (C) in BECs (red) or LECs (blue) cultured for 7 or 14 days under blood (B) or lymphatic (L) flow. The data represent mean ± SEM; n = 3. No statistical significance found between the experimental conditions; 2-way ANOVA, no statistically significant differences were observed. Symbols in bar graphs represent different experimental repeats with different donors (● repeat 1, ■ repeat 2, ▲ repeat 3).

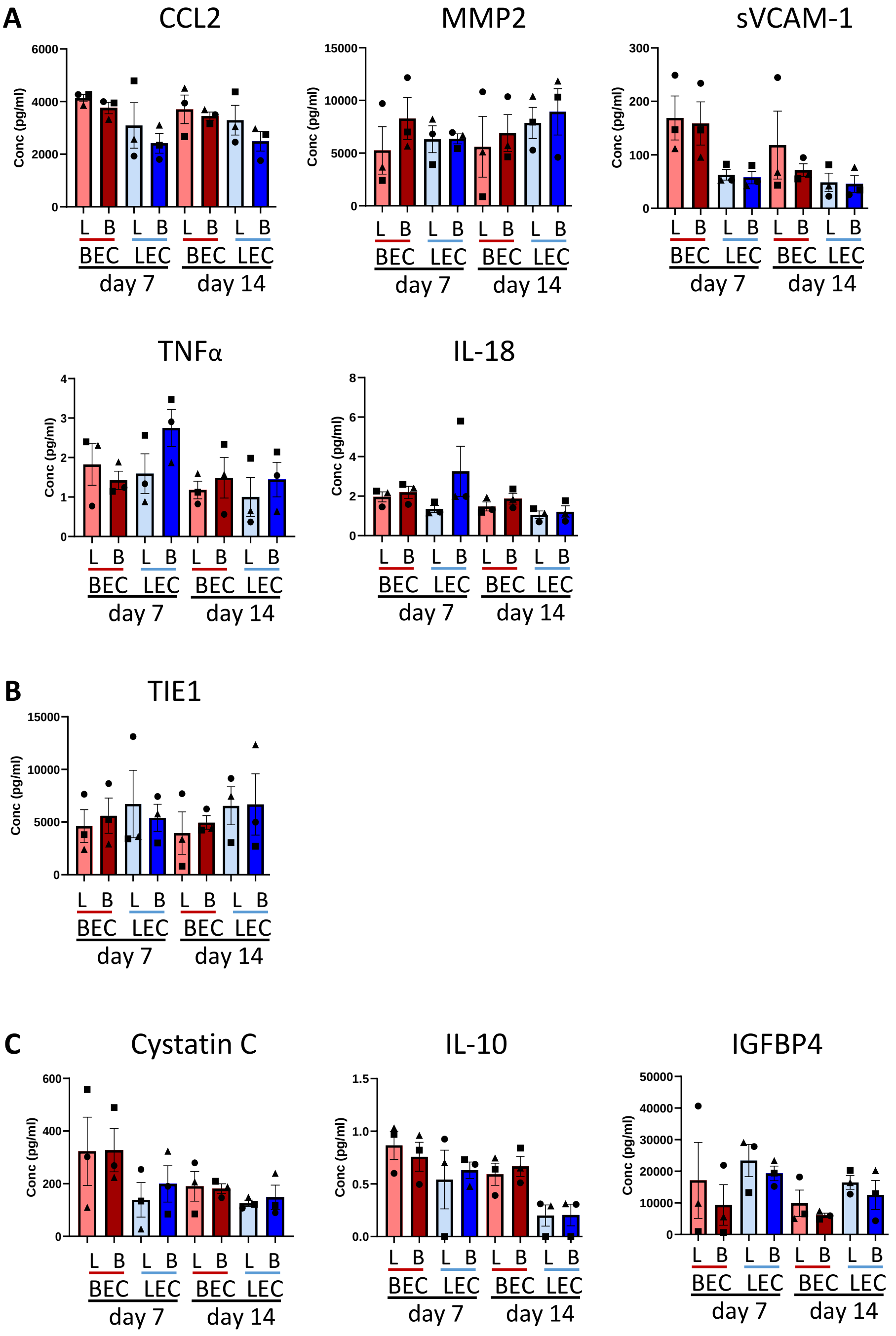

Supplementary Figure S2: BECs and LECs in a monolayer. Bright field images of BECs and LECs cultured with conventional 2D, static cell culture. Scale bar represents 200 µm.

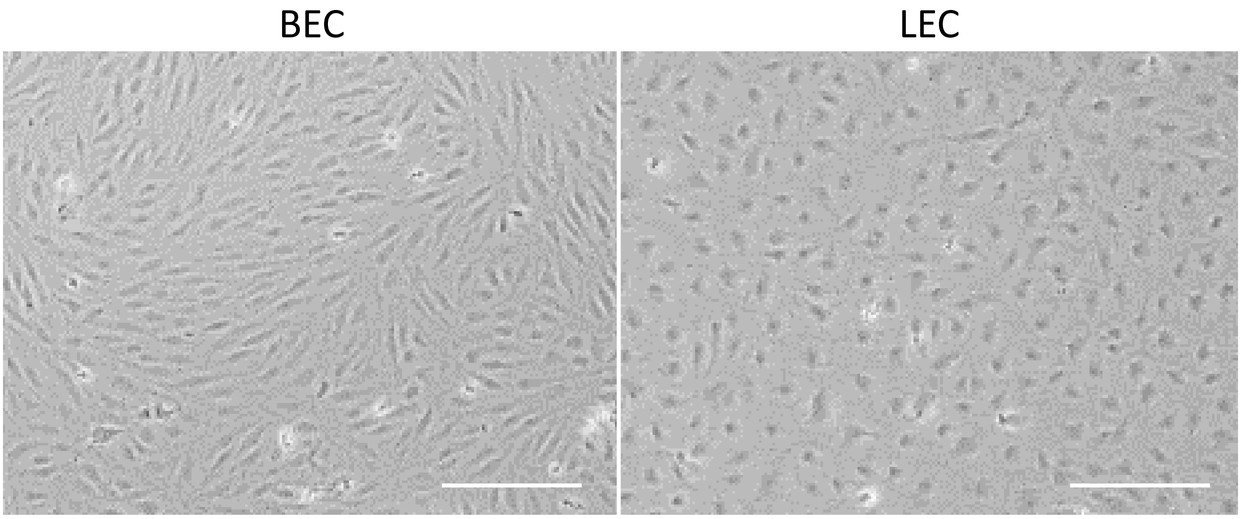

Supplementary Figure S3: Technical replicates for adherens and tight junction genes VE-cadherin, CLDN-5, ZO-1. Addition to Figure 5C.

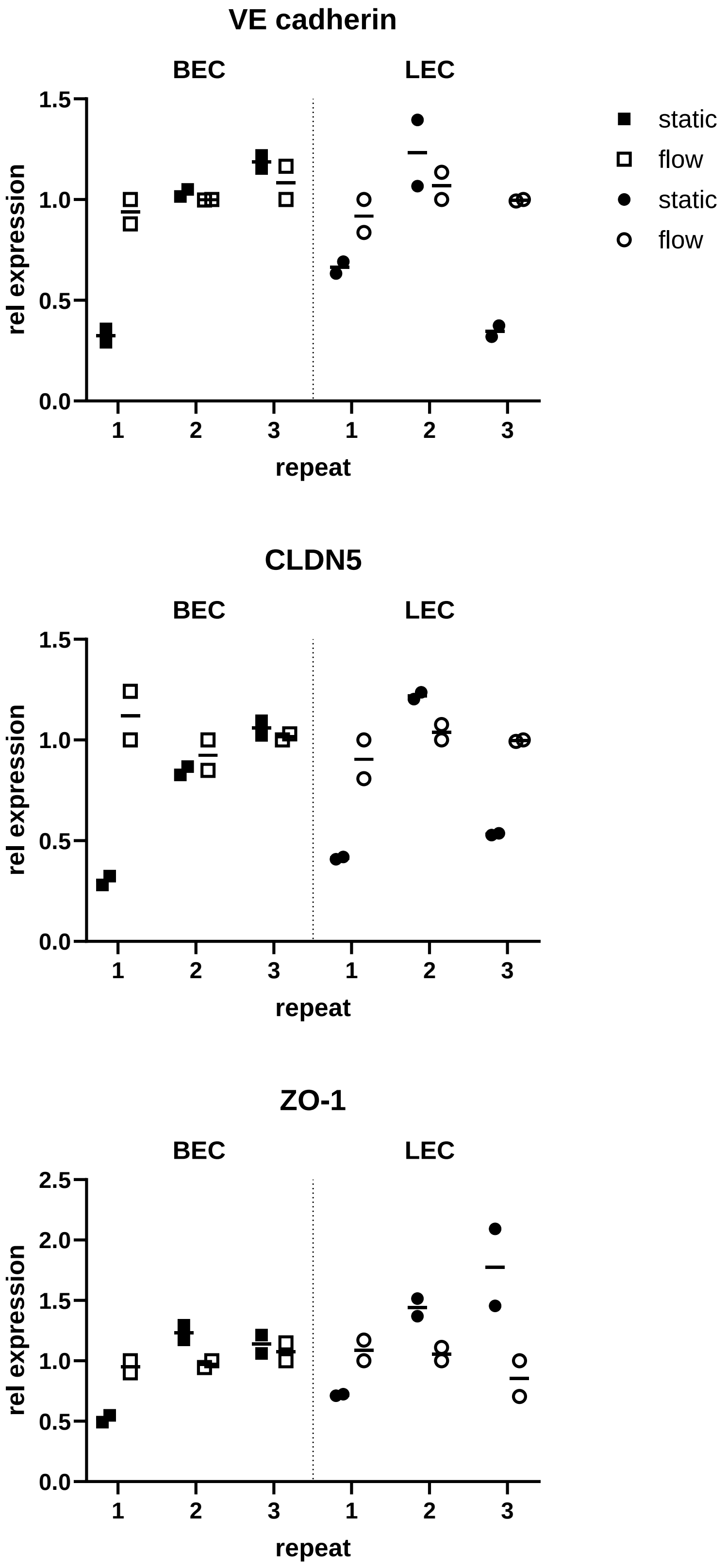

Supplementary Table 1: Primer sequences used.

Sequence	Name
**GTCTCCTCTGACTTCAACAGCG**	GAPDH fw
**ACCACCCTGTTGCTGTAGCCAA**	GAPDH rev
**CATTATGCTGAGGATTTGGAAAGG**	HPRT fw
**CTTGAGCACACAGAGGGCTACA**	HPRT rev
**AGCAGGAACCAAGCTTAGGCTG**	CCL21 fw
**GGTGTCTTGTCCAGATGCTGCA**	CCL21 rev
**CTGAAGACCTACTTCTCCGACG**	PROX1 fw
**GATGGCTTGACGTGCGTACTTC**	PROX1 rev
**TCCAGCTCTGCTGAGGAGTACG**	TFF3 fw
**ATCCTGGAGTCAAAGCAGCAGC**	TFF3 rev
**ATGTGGCAGGTGACCGCCTTC**	CLDN5 fw
**CGAGTCGTACACTTTGCACTGC**	CLDN5 rev
**GTCCAGAATCTCGGAAAAGTGCC**	ZO1 fw
**CTTTCAGCGCACCATACCAACC**	ZO1 rev
**ACGCCTTCATGGTGTGGGCAAA**	SOX18 fw
**GTTCAGCTCCTTCCACGCTTTG**	SOX18 rev
**TGCACGTTGACTCAGCCGAGTA**	COUPTF2 fw
**AAGCACACTGAGACTTTTCCTGC**	COUPTF2 rev
**GAAGCCTCTGATTGGCACAGTG**	VE-Cadherin fw
**TTTTGTGACTCGGAAGAACTGGC**	VE-Cadherin rev
